# Role of the Adiponectin Binding Protein, T-Cadherin (*Cdh13*), in Allergic Airways Responses in Mice

**DOI:** 10.1371/journal.pone.0041088

**Published:** 2012-07-17

**Authors:** Alison S. Williams, David I. Kasahara, Norah G. Verbout, Alexey V. Fedulov, Ming Zhu, Huiqing Si, Allison P. Wurmbrand, Christopher Hug, Barbara Ranscht, Stephanie A. Shore

**Affiliations:** 1 Molecular and Integrative Physiological Sciences Program, Department of Environmental Health, Harvard School of Public Health, Boston, Massachusetts, United States of America; 2 Children’s Hospital, Boston, Massachusetts, United States of America; 3 Sanford -Burnham Medical Research Institute, La Jolla, California, United States of America; 4 Harvard Medical School, Brigham and Women’s Hospital, Boston, Massachusetts, United States of America; Leiden University Medical Center, Netherlands

## Abstract

Adiponectin is an adipose derived hormone that declines in obesity. We have previously shown that exogenous administration of adiponectin reduces allergic airways responses in mice. T-cadherin (T-cad; *Cdh13*) is a binding protein for the high molecular weight isoforms of adiponectin. To determine whether the beneficial effects of adiponectin on allergic airways responses require T-cad, we sensitized wildtype (WT), T-cadherin deficient (T-cad^−/−^) and adiponectin and T-cad bideficient mice to ovalbumin (OVA) and challenged the mice with aerosolized OVA or PBS. Compared to WT, T-cad^−/−^ mice were protected against OVA-induced airway hyperresponsiveness, increases in BAL inflammatory cells, and induction of IL-13, IL-17, and eotaxin expression. Histological analysis of the lungs of OVA-challenged T-cad^−/−^ versus WT mice indicated reduced inflammation around the airways, and reduced mucous cell hyperplasia. Combined adiponectin and T-cad deficiency reversed the effects of T-cad deficiency alone, indicating that the observed effects of T-cad deficiency require adiponectin. Compared to WT, serum adiponectin was markedly increased in T-cad^−/−^ mice, likely because adiponectin that is normally sequestered by endothelial T-cad remains free in the circulation. In conclusion, T-cad does not mediate the protective effects of adiponectin. Instead, mice lacking T-cad have reduced allergic airways disease, likely because elevated serum adiponectin levels act on other adiponectin signaling pathways.

## Introduction

Obesity is a major public health issue [1]. Epidemiological data indicate that asthma prevalence is increased in obese individuals [2], [3], [4], [5], and that obesity antedates asthma. Furthermore, weight-loss improves asthma outcomes [6], [7]. The mechanistic basis for obesity-related asthma remains to be established.

Data from animal models suggest a role for alterations in the adipocyte-derived hormone, adiponectin, in the relationship between obesity and asthma. Adiponectin has anti-inflammatory properties. For example, adiponectin induces an M2, anti-inflammatory, phenotype in macrophages and inhibits LPS induced production of TNFα and MCP-1 [8], [9], [10], [11], [12], [13]. Adiponectin inhibits expression of adhesion molecules such as VCAM-1 in endothelial cells [14], [15] and also induces expression of the anti-inflammatory genes, IL-10 and the endogenous IL-1 receptor antagonist, IL-1RA [16]. Serum adiponectin declines in obesity and rises with weight loss, suggesting that loss of the anti-inflammatory actions of adiponectin in obesity could augment allergic inflammation. Exogenous administration of adiponectin inhibits allergic airways responses in mice, including airway hyperresponsiveness (AHR) and airway eosinophilia [17], [18]. Others have shown that adiponectin deficient mice (Adipo^−/−^) exhibit increased responses to chronic allergen challenge [19]. Epidemiological studies also support a beneficial role for adiponectin in asthma [20].

Three adiponectin binding proteins have been cloned; adipo-R1, -R2 and T-cadherin (T-cad) [21], [22], [23]. Lung tissue expresses all three proteins [21], [24], [25]. In the heart, the beneficial effects of adiponectin appear to require T-cad. Adiponectin associates with heart tissue in wildtype (WT) mice, but not in mice deficient in T-cad (T-cad^−/−^ mice). T-cad^−/−^ mice display increased cardiac hypertrophy following pressure overload and increased infarction size after ischemia-reperfusion, similar to Adipo^−/−^ mice [26]. Importantly, the protective effects of adiponectin in these models are not observed in T-cad^−/−^ mice, suggesting that T-cad mediates the effects of adiponectin. On the other hand, T-cad^−/−^ mice have substantially elevated serum adiponectin [26], [27], [28], [29] which would be expected to enhance effects of adiponectin mediated by other adiponectin binding proteins.

The purpose of this study was to determine the impact of T-cad deficiency on allergic airways responses. We sensitized and challenged WT and T-cad^−/−^ mice with ovalbumin (OVA) and measured OVA-induced airway hyperresponsiveness (AHR) and airway inflammation. To confirm that the observed effects of T-cad deficiency were the result of the adiponectin binding properties of T-cad, we also sensitized and challenged mice bideficient for adiponectin and T-cad (Adipo^−/−/^T-cad^−/−^) mice.

## Methods

### Animals

The following studies were approved by the Harvard Medical Area Standing Committee on Animals. WT, Adipo^−/−^, T-cad^−/−^, and Adipo^−/−/^T-cad^−/−^ mice were used in this study. T-cad^−/−^ mice were generated as previously described [28]. Adipo^−/−^ mice were obtained from Dr. Yuji Matsuzawa [30]. Adipo^−/−/^T-cad^−/−^ mice were generated by mating Adipo^−/−^ mice with T-cad^−/−^ mice, backcrossing the offspring with T-cad^−/−^ mice, and mating the resulting Adipo^+/−/^T-cad^−/−^ offspring. The resulting Adipo^−/−/^T-cad^−/−^ mice served as founders for the colony used in this study. Because the Adipo^−/−^ mice had been backcrossed to Jackson C57BL/6 mice, whereas the T-cad^−/−^ mice had been backcrossed to Taconic C57BL/6 mice, and because Taconic and Jackson mice differ in their colonic bacteria [31] which may affect allergic responses, we generated WT controls for these experiments by mating Jackson and Taconic C57BL/6 mice, then mating the offspring to Taconic C57BL/6. The resulting pups became the founders for the WT mice used as controls for the Adipo^−/−/^T-cad^−/−^ mice. A similar process was used to generate the T-cad^−/−^ mice that were compared to the Adipo^−/−/^T-cad^−/−^ mice. For comparison of WT and Adipo^−/−^ mice, C57BL/6J mice (Jackson Labs, Bar Harbor, ME) were used.

### Protocol

Male WT (C57BL/6 mice), T-cad^−/−^ mice, and Adipo^−/−/^T-cad^−/−^ mice were sensitized to chicken egg albumin (OVA, grade V; Sigma-Aldrich Co., St. Louis, MO) on Days 0 and 14 by i.p. injection of 20 µg of OVA and adjuvant, 2 mg of aluminum hydroxide (J.T. Baker, Phillipsburg, NJ) dispersed in 0.2 ml of PBS. On days 28 through 30, mice were challenged for 30 minutes with either aerosolized PBS or PBS containing 1% OVA (weight/volume) [32]. Twenty four hours after the last challenge, airway responsiveness to aerosolized methacholine was assessed, blood was collected, and bronchoalveolar lavage (BAL) was then performed. In an additional series of experiments, female WT, T-cad^−/−^, and Adipo^−/−/^T-cad^−/−^ mice were sensitized in the same way, but challenged on days 28 through 34 with 6% OVA or PBS, and examined 48 hours post exposure. For this series, two cohorts were used, one for airway responsiveness, BAL, tissue collection and the other for histology. Female WT and Adipo^−/−^ mice were also sensitized and challenged with 6% OVA in the same manner.

### Measurement of Pulmonary Mechanics and Airway Responsiveness

Mice were anesthetized with xylazine (7 mg/kg/i.p) and sodium pentobarbital (50 mg/kg/i.p), intubated with a tubing adaptor (18 gauge, Becton Dickinson), and ventilated (FlexiVent, SCIREQ, Montreal, Canada) with a tidal volume (V_T_) of 0.3 ml at 150 breaths/minute. Once ventilation was established, the chest wall was opened bilaterally to expose the lungs to atmospheric pressure. A positive end-expiratory pressure (PEEP) of 3 cm H_2_O was applied by placing the expiratory line under water. A standardized volume history was established by twice inflating the lungs to 30 cm H_2_O airway opening pressure (total lung capacity, TLC). In order to measure the static elastic properties of the lung, we then performed a slow inflation to TLC, followed by a slow deflation back to end expiratory lung volume. This quasi static pressure volume (PV) loop was repeated 3 times at 1 minute intervals for each PV loop, the Salazar Knowles equation [33] was used to determine the parameters A, B, and K. A is the difference between TLC and end expiratory lung volume, B is the difference between TLC and the extrapolated volume at zero transpulmonary pressure, and K is the curvature of the upper portion of the deflationary limb of the PV curve. Static compliance (Cstat) was obtained from the lower part of the deflationary limb of the PV loop. Values from the 3 PV loops were averaged to obtain a mean value for each animal. Pulmonary mechanics and responses to inhaled aerosolized methacholine (MCh) dissolved in PBS were then measured using the forced oscillation technique [34], [35] as follows. First, aerosolized PBS was delivered for 10 seconds using an ultrasonic nebulizer (Aeroneb, SCIREQ). Pulmonary resistance (R_L_) was measured using a sinusoidal forcing function at 2.5 Hz every 15 seconds for the next three minutes. This sequence was then repeated with doses of methacholine, increasing from 0.3 to 300 mg/ml. At each dose, the 3 highest values of R_L_ were averaged.

### Bronchoalveolar Lavage

Lungs were lavaged twice with 1 ml PBS and the lavagates pooled. BAL fluid was centrifuged and total BAL cells and differentials were assessed as previously described [29]. BAL supernatant was frozen at −80°C. Total BAL protein was determined using the Bradford technique (Bio-Rad; Hercules, CA). BAL adiponectin, IL-13, IL-17, eotaxin, IL-5, MCP-1, TNFα and MUC5AC were analyzed by ELISA (R&D Systems, eBioscience, or Biolegend, USA, and Cusabio, Wuhan, China (for MUC5AC).

### Blood Collection

Following euthanasia with pentobarbital sodium, blood was collected from the heart via right ventricle puncture. Serum was isolated and stored at −80°C until analyzed for adiponectin and total IgE, and OVA-specific IgE by ELISA (R&D Systems for adiponectin, Biolegend, USA for IgE, Cayman, USA for OVA-specific IgE).

### RNA Extraction and Real Time PCR

The lungs were excised and the left lung immersed in RNAlater (Qiagen) and used to prepare RNA using RNeasy columns (Qiagen) including a DNase II step to remove genomic DNA. 2 µg of RNA was converted into cDNA using oligodT and “First-strand amplification kit” (Invitrogen, USA). T-cad, AdipoR1 and AdipoR2 mRNA were quantified using real time PCR (7300 Real-Time PCR Systems, Applied Biosystems, US) with primers previously described [17] and SYBR-green detection. Gene expression values were normalized to 18S and expressed relative to WT PBS values, using the delta Ct (ΔCt) method [36].

### Histological Assessment of Pulmonary Inflammation and Mucous cell Hyperplasia

Lungs were inflated with 10% formalin at 20 cmH_2_O pressure. The left lung was embedded in paraffin, sectioned, and stained with hematoxylin and eosin to determine the inflammation index (a product of the severity and prevalence of airway inflammation) using a modification of the method of Hamada *et* *al* [37]. Severity was assigned a numerical value based upon the number of inflammatory cell infiltrate layers around an airway (0, no cells; 1, ≤3 cell layers; 2, 4–9 cell layers; 3, ≥10 cell layers). Prevalence was assigned a numerical value according to the percentage of the circumference encompassed by inflammatory cells (0, none; 1, ≤25%; 2, 25–50%; 3, >50%). In each mouse, a minimum of 8 airways was examined and a mean value for each mouse computed. Other sections were stained with periodic acid-Schiff (PAS) to identify the prevalence of mucus-containing goblet cells. For each airway, a mucous hyperplasia index was assigned as follows: 0, no PAS staining cells; 1, ≤25%; 2, 25–50%; 3, >50% of the airway circumference consisted of PAS staining cells.

### Dendritic Cell/T-lymphocyte Co-cultures

To determine whether adiponectin impacts dendritic cell activation of T-lymphocytes, spleens were harvested from DO.11 mice (Jackson Labs, Bar Harbor, ME). These transgenic mice express the mouse alpha-chain and beta-chain T-cell receptor that pairs with the CD4 coreceptor and is specific for chicken OVA 323–339 peptide in the context of I-A b. In these mice, T-cells demonstrate a dose-dependent proliferative response to the specific OVA ligand. T-cells from DO.11 mice and dendritic cells from Balb/c mice were isolated from splenocyte suspensions and co-cultured as previously described [32], [38]. To test the allergen presentation activity of dendritic cells OVA protein (Sigma) was added to the cultures; control wells were left unstimulated. Tritiated thymidine (Sigma) was added to assess lymphocyte proliferation in response to OVA. Cells were washed 48 hours later to remove unincorporated thymidine, lysed, and radioactivity counted. In some wells, full length or trimeric (C22A) murine recombinant adiponectin, generated as described below, was added 24 hours prior to OVA stimulation. Control wells were treated with the TBS++ buffer used in which adiponectin was dissolved.

### Preparation of Full Length or C22A Adiponectin

cDNA encoding full length murine adiponectin or C22A adiponectin, each with a 3′-Flag tag, was cloned into the pcDNA3.1(+) mammalian expression vector (Invitrogen, Carlsbad, CA) as previously described [22] and transfected into HEK 293 cells gifted from Dr C Hug [22] using Lipofectamine 2000 (Invitrogen). Cys22 is required for formation of the hexameric and hexameric and high molecular weight (HMW) adiponectin oligomers, and replacing the cysteine at position 22 with an alanine results in adiponectin that exists as a single trimeric species, while full length adiponectin generates the trimeric as well as the HMW isoforms [39]. Transfection medium was removed after 24 hours, and replaced with serum-free OptiMEM I (Invitrogen) supplemented with 100 mg/L ascorbic acid, 55.5 mg/L CaCl_2_, and 500 mg/L sodium bicarbonate, pH 7.4. Conditioned medium containing the secreted adiponectin was collected every 48 hours and replaced with fresh supplemented OptiMEM I; a total of 900 mL was collected representing three collection pools. Collected medium was centrifuged at 1000 rpm for ten minutes at 4°C to remove cellular debris and stored at 4°C. For purification using the Flag tag, pooled conditioned medium was mixed with 4 mL packed volume M2 Anti-Flag Agarose prepared per manufacturer (Sigma-Aldrich, St. Louis, MO) overnight at 4°C with gentle rocking and collected using a column. Unbound protein was removed by extensive washing (10 bed volumes) with TBS++ (25 mM Tris, 147 mM NaCl, 2.7 mM KCl, 0.9 mM CaCl_2_, 0.5 mM MgCl_2_, pH 7.4). Bound adiponectin was eluted in 0.5 mL fractions using three bed volumes of elution buffer (TBS++) containing 250 ug/mL free Flag peptide. Fractions containing purified adiponectin were identified by SDS-PAGE and Coomassie staining, pooled and dialyzed extensively against TBS++ buffer at 4°C (Pierce Slide-a-Lyzer Casette 10kDa MWCO). The dialysate was concentrated and analyzed for purity by silver stain and immunoblot using antibodies against adiponectin and the Flag epitope (Sigma), and protein concentration was determined using BCA protein assay kit (Pierce). Purified protein was stored at 4°C until use.

### Statistical Analysis

Comparisons of pulmonary mechanics, BAL, serum, histology, and mRNA expression were assessed by factorial analysis of variance (ANOVA), using genotype and challenge as main effects. Fisher’s least significant difference test was used as a follow-up test. Statistical analysis was performed using Statistica software (StatSoft, Inc.; Tulsa, OK). The results are expressed as mean ± SEM. A *p* value less than 0.05 was considered significant.

## Results

**Figure 1:**
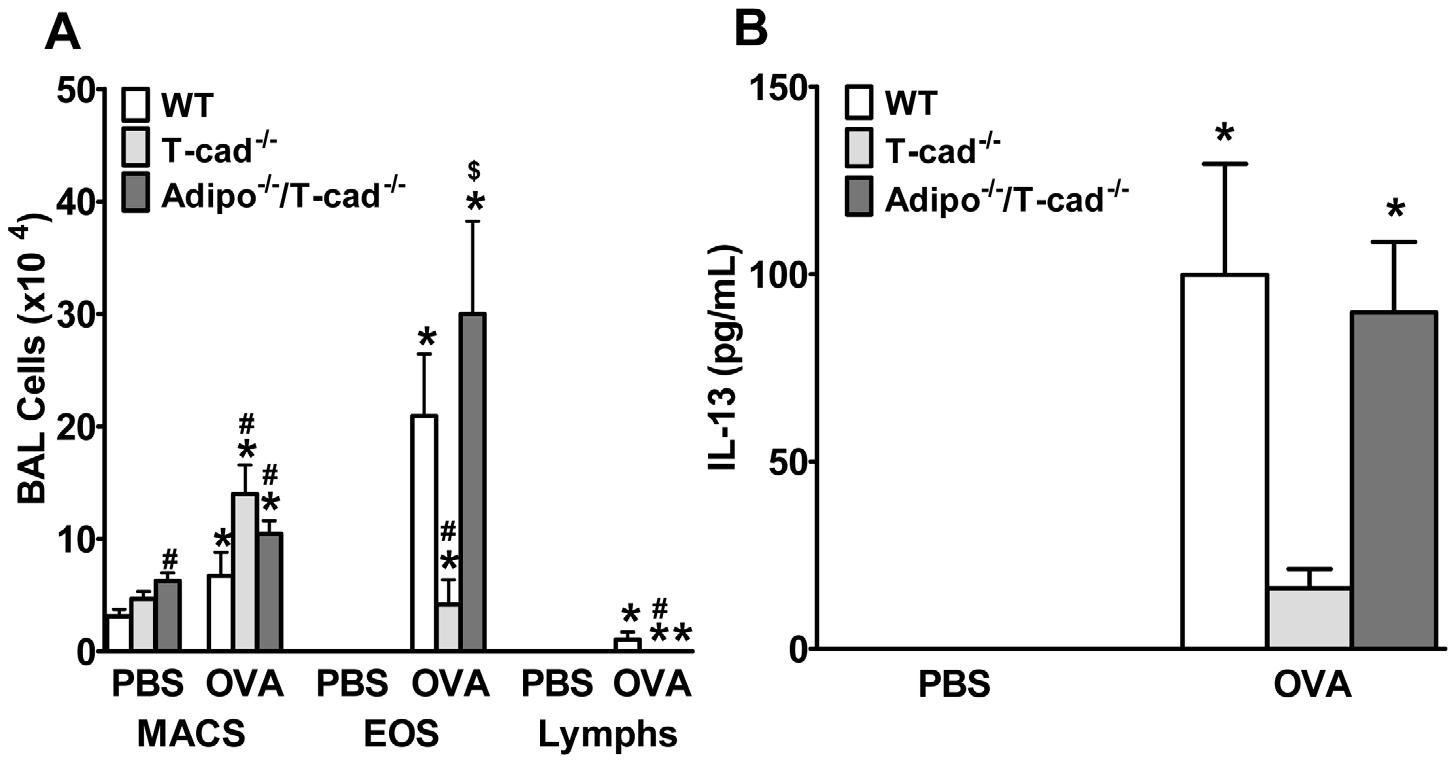
Impact of T-cad deficiency on allergic airways responses. We measured BAL cells (A) and IL-13 (B) in wildtype (WT), T-cadherin deficient (T-cad^−/−^), and dual adiponectin and T-cad deficient (Adipo^−/−/^T-cad^−/−^) mice challenged with 1% ovalbumin (OVA) or PBS. Results are mean±SEM, n = 7−9. *p<0.05, vs genotype-matched, PBS-challenged mice; #*p*<0.05, vs OVA- challenged WT. $*p*<0.05, vs OVA-challenged T-cad^−/−^.

In WT mice, OVA challenge (1% for 3 days) significantly increased BAL eosinophils, lymphocytes, and neutrophils (Figure 1A). In T-cad^−/−^ mice, this response was significantly decreased. Consistent with these observations, OVA-induced increases in BAL IL-13 were significantly reduced in T-cad^−/−^ versus WT mice (Figure 1B). These results are opposite in direction to what would be expected if T-cad was required for mediating the effects of adiponectin. T-cad is a member of the cadherin family of cell-cell adhesion molecules, and is implicated in cell migration, proliferation, adhesion, and survival. Hence, the observed effects of T-cad deficiency on allergic airways responses (Figure 1) may be unrelated to its adiponectin binding ability. To address this possibility, we examined mice deficient in both T-cadherin and adiponectin (Figure 1). BAL eosinophils and BAL IL-13 were significantly greater in Adipo^−/−/^T-cad^−/−^ mice than in T-cad^−/−^ mice, but were not significantly different from WT mice. Thus, combined T-cad and adiponectin deficiency reversed the effects of T-cad deficiency alone. The results indicate that adiponectin is required for the protective effects of T-cad deficiency in allergic airways inflammation.

**Figure 2:**
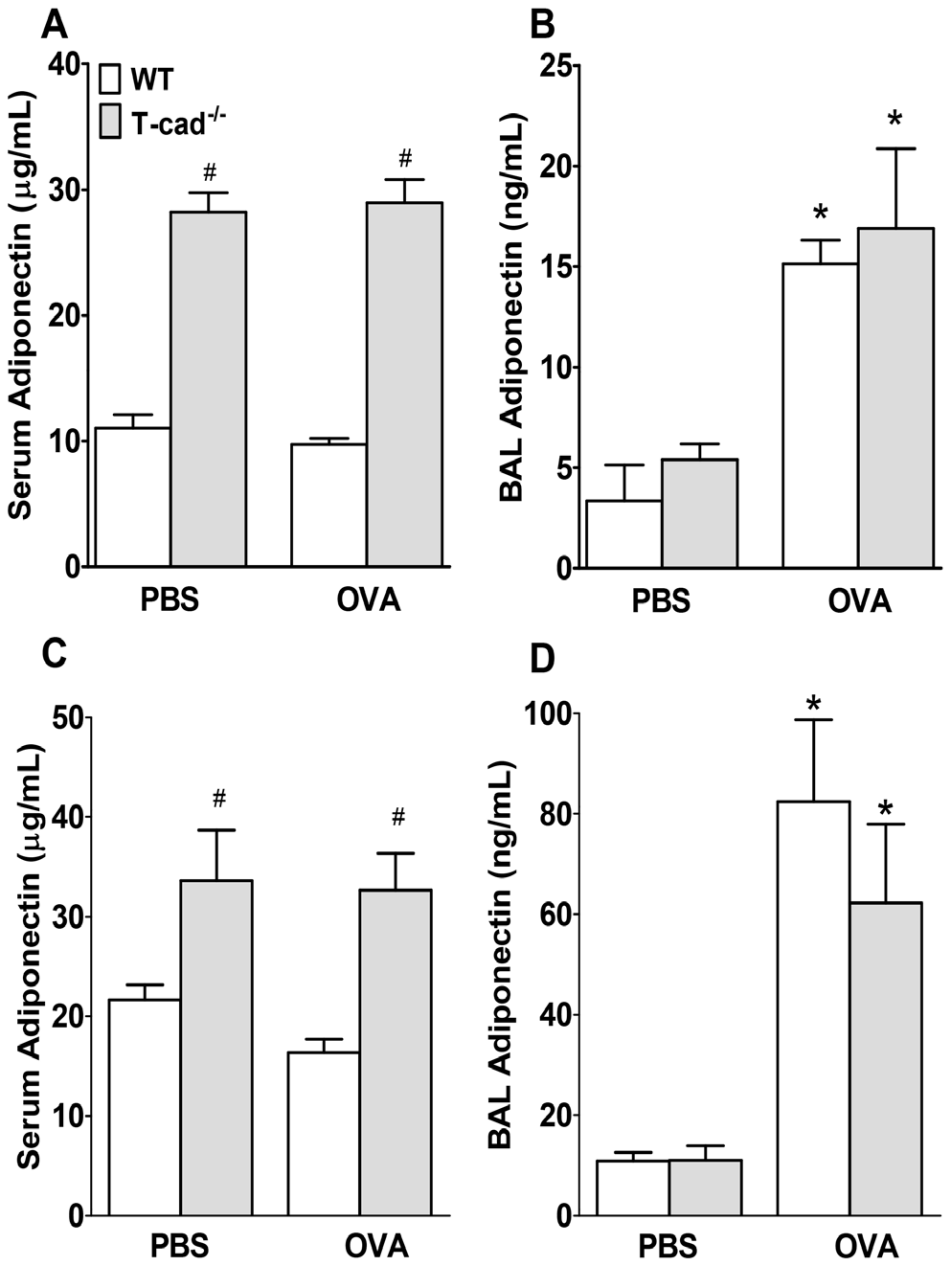
Impact of T-cad deficiency on BAL and serum adiponectin. Adiponectin levels in serum (A,C) and BAL (B,D) from 1% (A,B) or 6% (C,D) OVA or corresponding PBS-challenged WT and T-cad^−/−^ mice. Mice used in the 1% and 6% OVA experiments were male and female mice, respectively, which accounts for the greater serum adiponectin in the mice used in the 6% OVA than the 1% OVA challenge protocol. Results are mean±SEM. #p<0.05 vs genotype matched PBS mice; *p<0.05, vs exposure matched WT mice.

Serum adiponectin was almost 3-fold higher in T-cad^−/−^ versus WT mice (Figure 2A) under baseline conditions (PBS challenge). No changes were observed in response to OVA versus PBS challenge. BAL adiponectin was significantly greater in OVA versus PBS challenged mice (Figure 2B), with no notable differences between genotypes. Note that BAL adiponectin was substantively lower than serum adiponectin (ng/ml vs µg/ml respectively) regardless of exposure.

**Figure 3:**
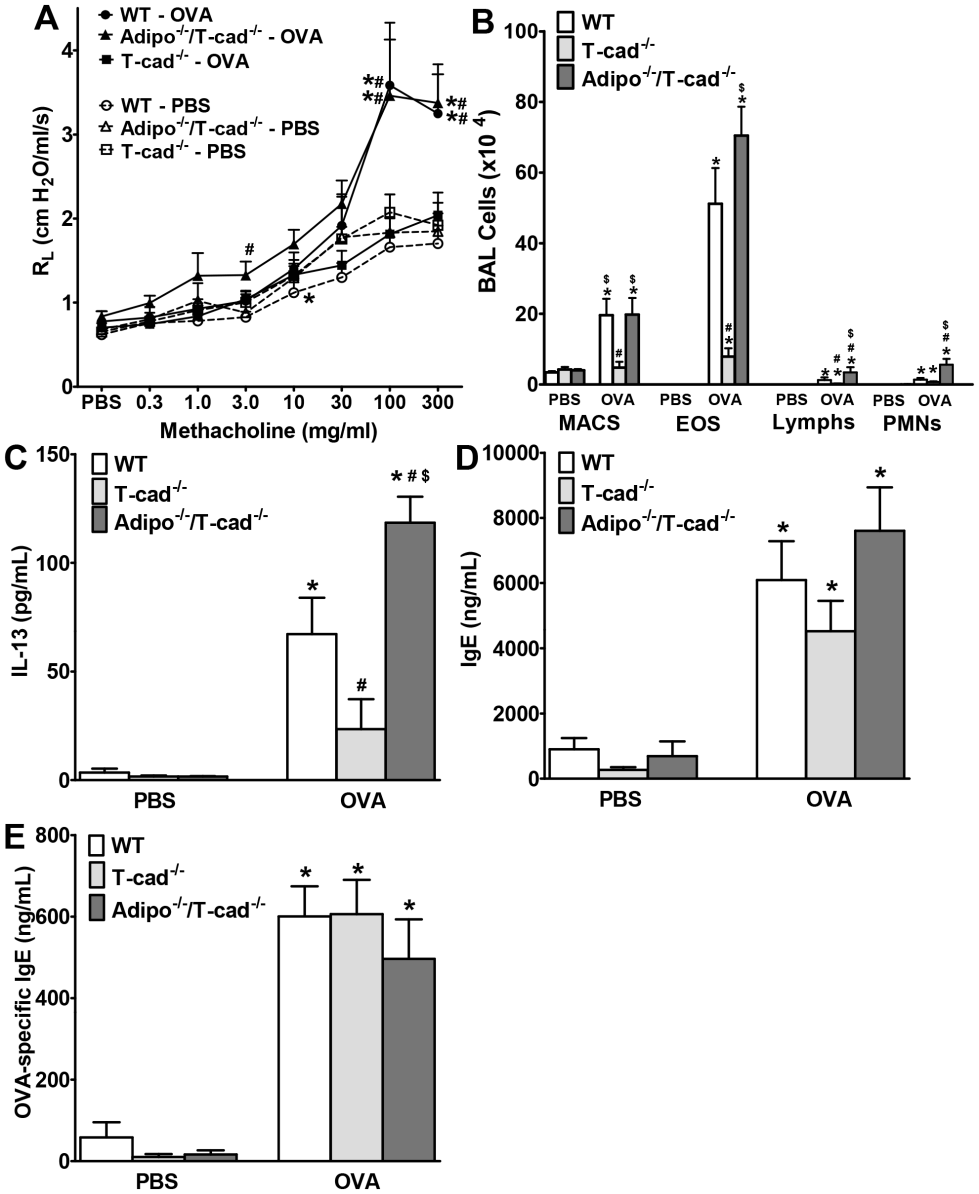
Effect of dual adiponectin and T-cad deficiency on allergic airways responses. We measured airway responsiveness (A), BAL cells (B), BAL IL-13 (C), serum total IgE (D) and serum OVA-specific IgE (E) in WT, T-cad^−/−^ and Adipo^−/−/^T-cad^−/−^ mice challenged with 6% OVA or PBS. Results are mean±SEM, n = 6−9. *p<0.05, vs genotype-matched, PBS-challenged mice; #*p*<0.05, vs OVA-challenged WT. $*p*<0.05, vs OVA-challenged T-cad^−/−^.

Consistent with the difficulties in inducing AHR by OVA challenge in C57BL/6 mice reported by others [40] we did not observe AHR following OVA challenge in WT, T-cad^−/−^, or Adipo^−/−/^T-cad^−/−^ mice using this 1% OVA challenge protocol, whether we used R_L_, dynamic compliance (Cdyn), airway resistance, or the coefficients of tissue damping or elastance (assessed as previously described) [41] as the outcome indicator. Consequently, we assessed airway responsiveness using a more intense OVA challenge (6% for 6 days). In WT mice, this latter OVA challenge protocol did cause AHR (Figure 3A). Moreover, OVA-induced AHR was abolished in T-cad^−/−^ mice, but not in Adipo^−/−/^T-cad^−/−^ versus WT mice. Note that airway responsiveness was similar in WT, T-cad^−/−^, and Adipo^−/−/^T-cad^−/−^ mice challenged with PBS.

Because unexposed Adipo^−/−^ mice have been reported to develop emphysema as they age [25], [29], [42], [43] which could affect measurements of airway responsiveness, we also examined parameters describing the PV curve of the lung (Table 1) of the PBS challenged mice. Emphysema would be expected to increase both lung volumes (A and B) and Cstat. However, we observed no significant effect of either T-cad deficiency or bideficiency in adiponectin and T-cad on the lung PV curve (Table 1). The mice were quite young (9 weeks of age) and others have also reported very limited changes in total lung capacity in Adipo^−/−^ mice at 8 weeks of age, whereas TLC is significantly elevated in Adipo^−/−^ mice at 30 weeks of age [25].

**Table 1:** PV curve parameters of the lungs of female mice challenged with PBS.

Parameter	WT	T-cad^−/−^	Adipo^−/−/^T-cad^−/−^
A (ml)	0.61±0.04	0.67±0.05	0.65±0.05
B (ml)	0.99±0.05	1.07±0.09	1.01±0.09
K (cm H_2_O^−1^)	0.145±0.004	0.142±0.004	0.132±0.003
Cstat (ml/cm H_2_O)	0.070±0.004	0.075±0.006	0.069±0.006

Results are mean ± SE of data from 6–7 mice in each group. A, B, and K were derived using the Salazar-Knowles equation [33]; Cstat: static compliance.

**Figure 4:**
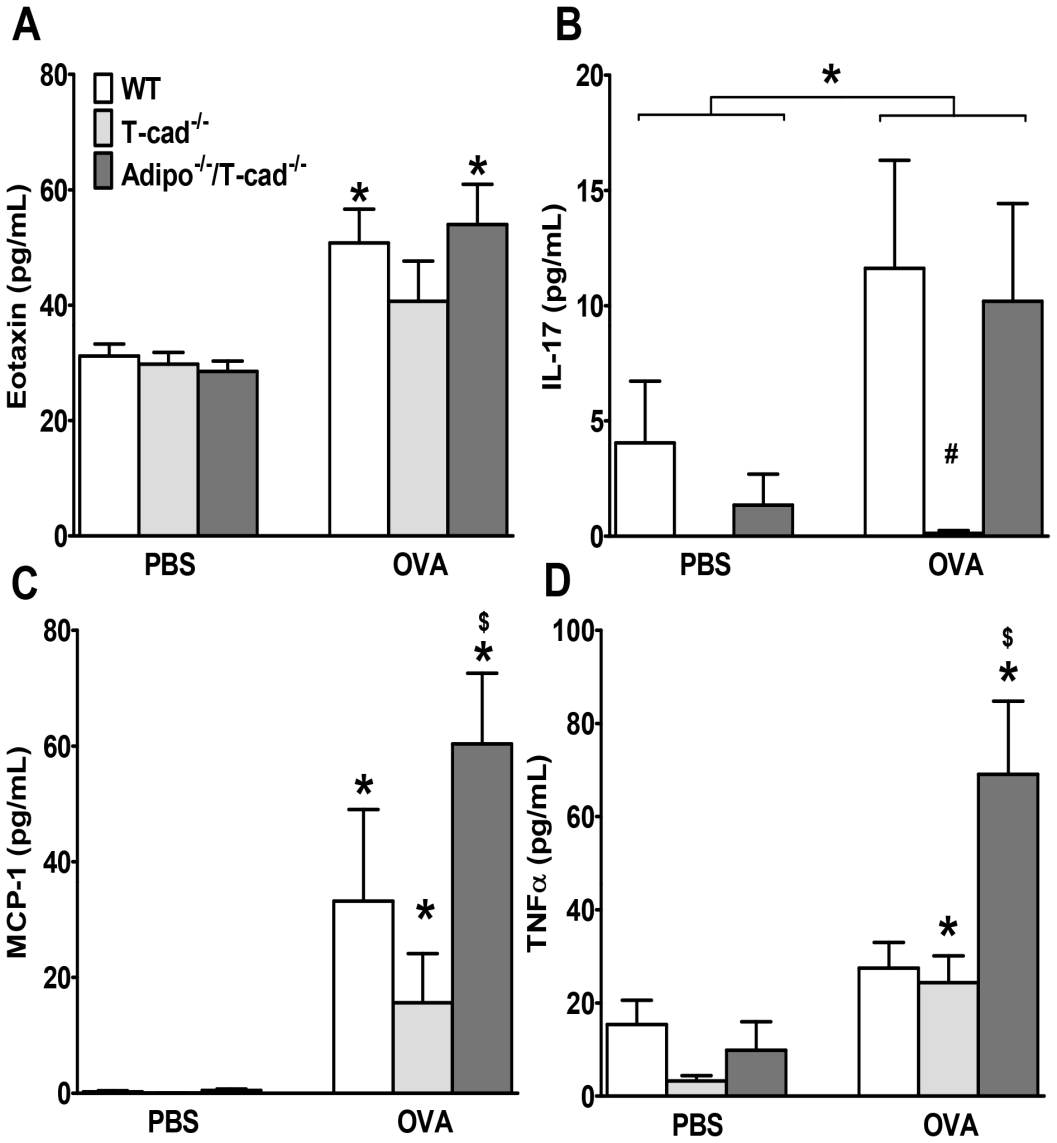
Effect of dual adiponectin and T-cad deficiency on cytokine and chemokine expression. BAL eotaxin (A), IL-17 (B), MCP-1 (C), and TNFα (D) in WT, T-cad^−/−^ and Adipo^−/−/^T-cad^−/−^ mice challenged with 6% OVA or PBS. Results are mean±SEM, n = 3 for PBS mice, n = 7 for OVA mice. *p<0.05 vs genotype-matched, PBS-challenged mice; #*p*<0.05, vs OVA-challenged WT. $*p*<0.05, vs OVA-challenged T-cad^−/−^.

The impact of T-cad deficiency on BAL inflammatory parameters was similar using this 6% OVA challenge protocol as it was with the 1% protocol: OVA challenge caused significantly greater increases in BAL eosinophils, lymphocytes, and IL-13 in WT versus T-cad^−/−^ mice, and combined adiponectin and T-cad deficiency reversed the effects of T-cad deficiency alone (Figures 3B, C). Indeed, BAL lymphocytes, neutrophils, and IL-13 were actually elevated above WT in Adipo^−/−/^T-cad^−/−^ mice. OVA challenge caused a marked increase in serum total IgE and OVA-specific IgE in mice of all three genotypes, but there was no effect of genotype on IgE levels (Figure 3D, E). IL-5 was at or below the limit of detection of the ELISA in most mice (data not shown). Factorial ANOVA indicated a significant effect of both OVA exposure and mouse genotype on BAL eotaxin. Compared to PBS treated mice, BAL eotaxin was increased in OVA exposed WT and Adipo^−/−/^T-cad^−/−^ but not T-cad^−/−^ mice (Figure 4A). Given the importance of eotaxin for eosinophil recruitment in allergic airways disease [44] the reduction in eotaxin may account for the decline in BAL eosinophils in the T-cad^−/−^ mice (Figures 1A, 3B). There was also a significant effect of both OVA challenge and mouse genotype on BAL IL-17 (Figure 4B). IL-17 was significantly greater in WT than T-cad^−/−^ OVA exposed mice. IL-17 was also greater in Adipo^−/−/^T-cad^−/−^ versus T-cad^−/−^ OVA exposed, although the effect did not quite reach statistical significance (p<0.07). Given the important role of IL-17 in neutrophil recruitment [45], IL-17 may account for some of the genotype related differences in neutrophil recruitment observed in these mice (Fig. 1A, 3B).

As was the case with the 1% OVA challenge protocol, BAL adiponectin was significantly greater in mice challenged for 6 days with 6% OVA than in PBS challenged mice (Figure 2D), but there was no effect of genotype on BAL adiponectin. Serum adiponectin was significantly greater in the T-cad^−/−^ than the WT mice (Figure 2C) regardless of challenge.

Because both MCP-1 (CCL2) and TNFα have been shown to contribute to aspects of allergic airways disease in mice [46], [47], and because expression of both MCP-1 and TNFα is suppressed by adiponectin in cultured macrophages [9], [13], we also measured BAL MCP-1 and TNFα in these mice (Figures 4C, D). We observed a different genotype-dependent pattern of changes in MCP-1 and TNFα, versus the other cytokines and chemokines examined. OVA-induced increases in MCP-1 and TNFα were not different in WT and T-cad^−/−^ mice, but both cytokines were increased to a greater extent in Adipo^−/−/^T-cad^−/−^ than in T-cad^−/−^ mice (Figures 4C, D). Although the data provide evidence of a role for adiponectin in regulating TNFα and MCP-1 expression in lung during allergic inflammation, they also suggest that changes in TNFα and MCP-1 do not account for differences in inflammatory cell recruitment or airway hyperresponsiveness observed in WT vs T-cad^−/−^ mice.

**Figure 5:**
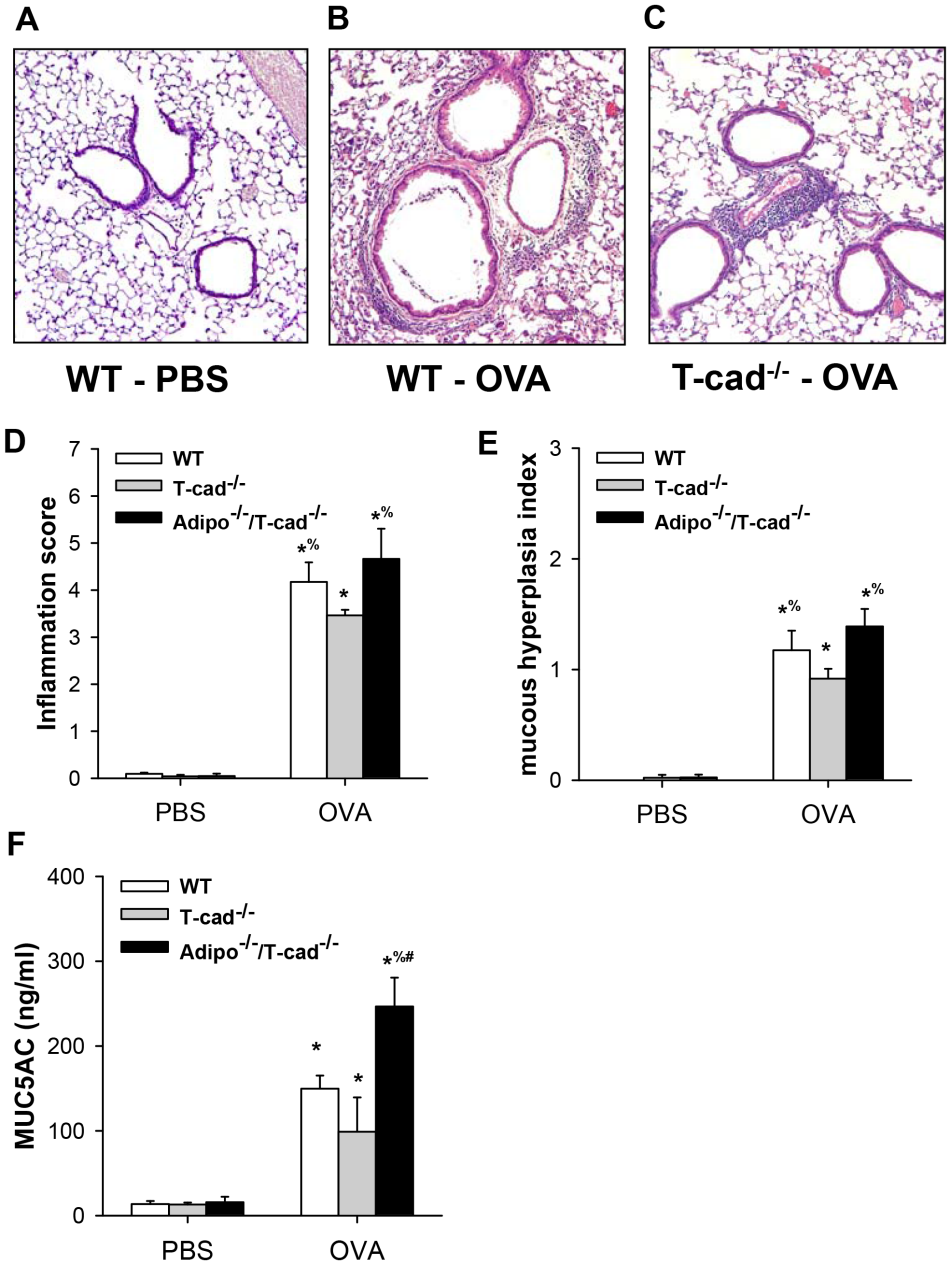
Histological assessment of inflammation and mucous cell hyperplasia. H&E stained sections of PBS-challenged WT (A), 6% OVA-challenged WT (B) and 6% OVA-challenged T-cad^−/−^ mice (C). Inflammation index for airways (D), mucous hyperplasia index (E), and BAL MUC5AC (F). Results are mean±SEM of 3–5 mice per group in the PBS treated groups and 5–6 mice per group in the OVA treated groups. *p<0.05 vs genotype-matched PBS-challenged mice; % *p*<0.05 vs exposure-matched T-cad^−/−^ mice; #*p*<0.05, vs OVA-challenged WT.

Histology confirmed an increase in inflammatory cells around the airways after OVA exposure (Figure 5). We did not observe any significant differences in inflammation in Adipo^−/−/^T-cad^−/−^ versus WT mice. However, airway inflammation was significantly reduced in T-cad^−/−^ mice versus both WT and Adipo^−/−/^T-cad^−/−^ mice (Figure 5D). Mucous cell hyperplasia, a key feature of asthma [48], was also induced by OVA (Figure 5E). The mucous hypersecretion index was significantly lower in T-cad^−/−^ versus both WT and Adipo^−/−/^T-cad^−/−^ mice, whereas WT and Adipo^−/−/^T-cad^−/−^ were not different. Compared to PBS, OVA challenge also induced a significant release of MUC5AC in BAL fluid (Fig. 5F). BAL MUC5AC was greater in Adipo^−/−/^T-cad^−/−^ than in either T-cad^−/−^ or WT mice. BAL MUC5AC was also greater in WT versus T-cad^−/−^ mice, but the effect did not reach statistical significance.

**Figure 6:**
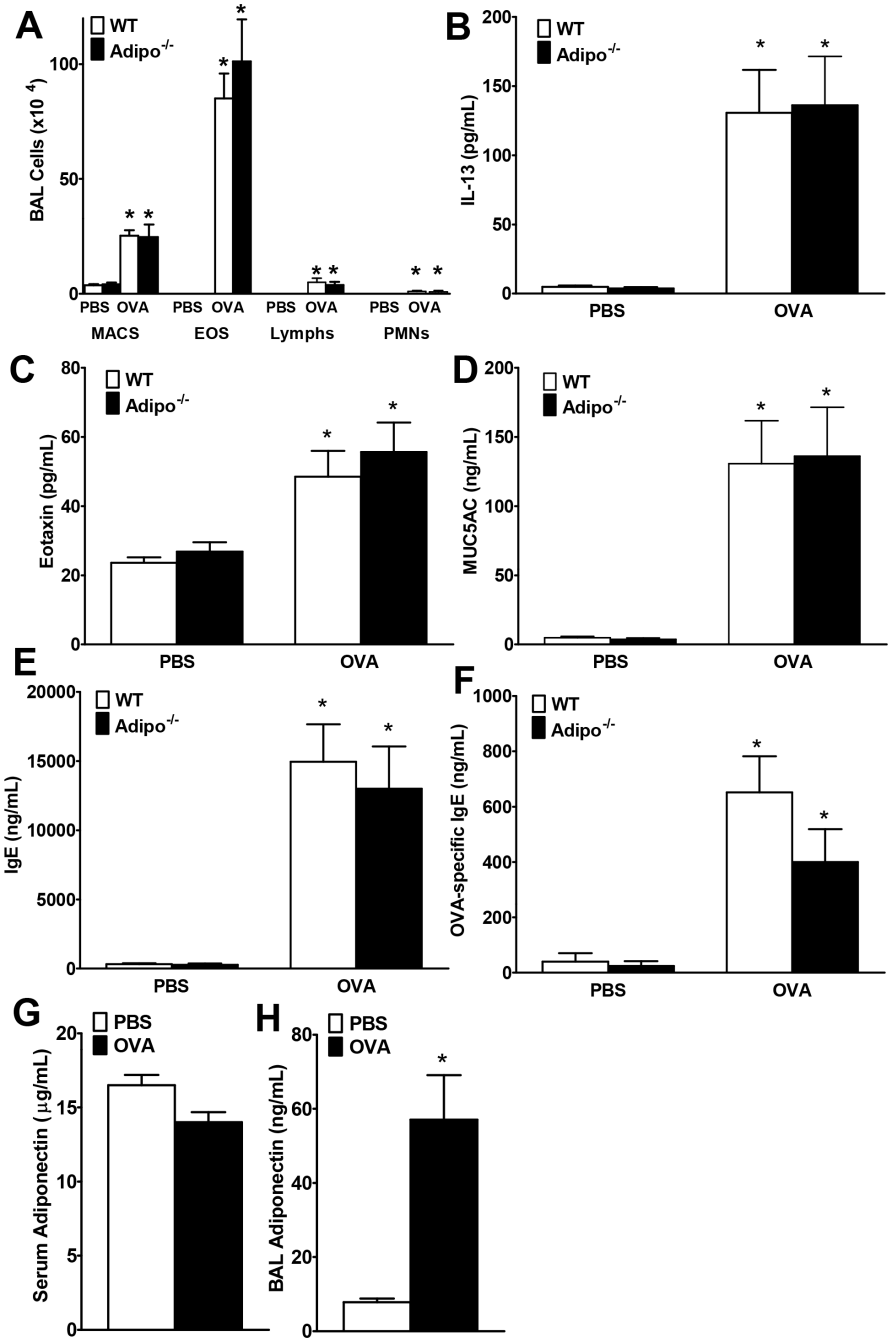
Effect of Adiponectin deficiency on allergic airways responses. BAL cells (A), BAL IL-13 (B), BAL eotaxin (C) BAL MUC5AC (D), serum total IgE (E) and serum OVA-specific IgE (F) in 6% OVA- or PBS-challenged Adipo^−/−^ or WT mice. Serum (G) and BAL (H) adiponectin from the WT mice are also shown. Results are mean±SEM, n = 3−8 mice/group, *p<0.05 vs genotype-matched, PBS-challenged mice; *#*<0.05 vs WT, OVA-challenged mice.

For comparison, we also examined allergic airways inflammation in WT and Adipo^−/−^ mice (Figure 6). Compared to PBS, OVA increased BAL eosinophils and lymphocytes, but there was no effect of adiponectin deficiency on any BAL cell type (Figure 6A). Similarly, deficiency in adiponectin alone did not impact OVA induced increases in BAL eotaxin (Figure 6C), BAL MUC5AC (Figure 6D), or either serum total IgE or OVA-specific IgE (Figures 6E, F). BAL adiponectin levels were increased from baseline (PBS-challenge) in WT mice after OVA-challenge, but not in serum (Figure 6G, H).

**Figure 7:**
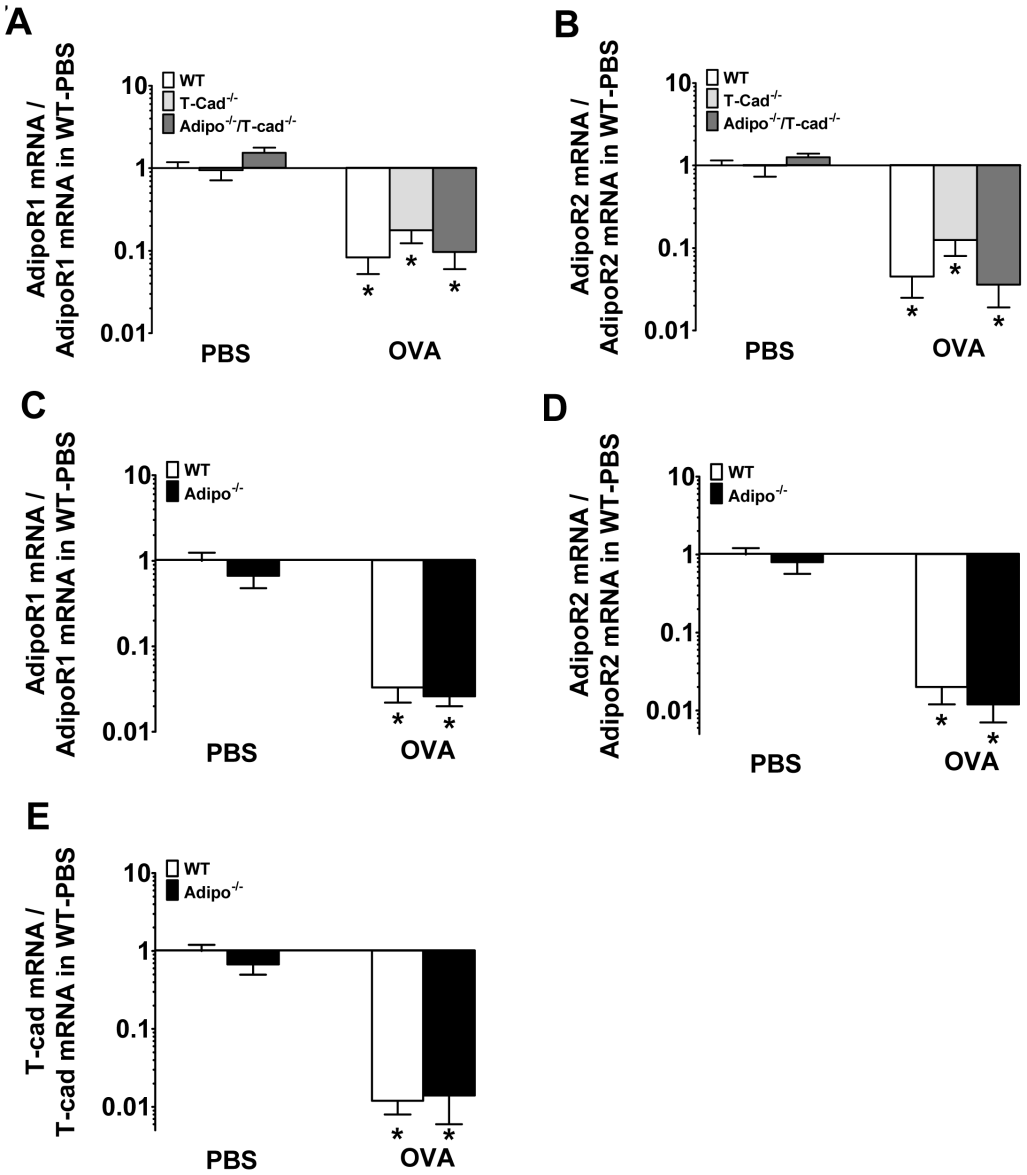
Lung mRNA expression of adiponectin binding proteins, AdipoR1, AdipoR2, and T-cadherin in allergic lungs. mRNA expression for AdipoR1 and AdipoR2 in 6% OVA- or PBS-challenged WT, T-cad^−/−^, and Adipo^−/−/^T-cad^−/−^ mice (A, B) and WT and Adipo^−/−^ mice (C, D), mRNA expression of T-cadherin in WT and Adipo^−/−^ mice (E). Results are mean±SEM, n = 6−8 mice/group. *p<0.05 vs genotype-matched, PBS-challenged mice.

Compared to PBS, OVA challenge decreased the expression of the adiponectin binding proteins, AdipoR1 and AdipoR2, in all strains of mice (Figure 7A–D). OVA challenge also decreased the expression of T-cad in both WT and Adipo^−/−^ mice (Figure 7E) consistent with the hypothesis that changes in levels of these adiponectin binding proteins contribute to allergic airways disease.

**Figure 8:**
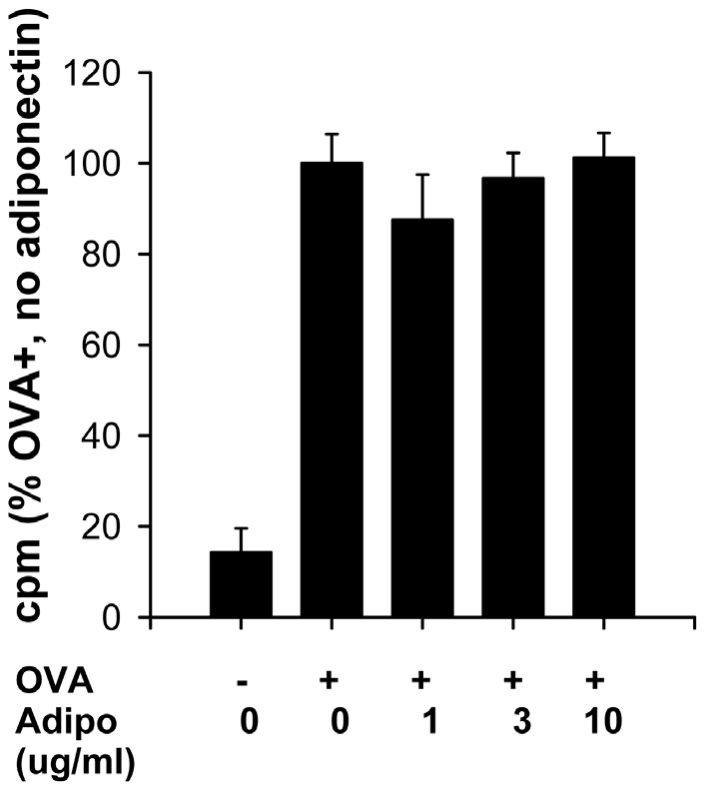
Effect of full length adiponectin on T-cell proliferation induced by culture with OVA stimulated dendritic cells. T-cells isolated from spleens of OVA-TCR transgenic mice were incubated with dendritic cells from WT mice and stimulated with OVA. Tritiated thymidine was added to assess cell proliferation. “0” adiponectin wells were treated with the vehicle (TBS++) used to dissolve adiponectin. CPM = counts per minute. Results are mean ± SEM of data from 6 wells in each condition. Data are expressed as a % of the OVA treated wells that received only vehicle.

To determine whether adiponectin might be acting at the level of dendritic cell presentation of antigens to T cells, we cultured dendritic cells with T-lymphocytes from DO.11 mice that are transgenic for a T-cell receptor that recognizes OVA peptide. Addition of OVA caused a robust increase in lymphocyte proliferation (Figure 8). However, full length adiponectin (which contains the trimeric, hexameric and HMW isoforms) [39] had no effect on OVA induced T cell proliferation (Figure 8). Trimeric adiponectin was also without effect (data not shown).

## Discussion

T-cad^−/−^ mice have markedly elevated blood adiponectin (Figure 2A, C and [26], [28], [29]). Results presented in this study support the hypothesis that this increase in serum adiponectin is protective against allergic airways responses. Allergic airways responses were reduced in T-cad^−/−^ versus WT mice (Figures 1, 3, 4, 5). Furthermore, dual adiponectin and T-cadherin deficiency reversed the effects of T-cad deficiency alone (Figures 1, 3, 4, 5) indicating that the observed effects of T-cad deficiency require adiponectin.

The purpose of this study was to determine whether T-cad, one of three adiponectin binding proteins, plays a role in pulmonary responses to allergens. T-cadherin is required for adiponectin to bind to endothelial cells [28] and cardiomyocytes [26]. Furthermore, the protective effects of adiponectin against cardiac hypertrophy induced by pressure overload and against cardiac injury induced by ischemia-reperfusion appear to require T-cad [26]. Given that T-cad mainly binds the hexameric and HMW isoforms of adiponectin [22], and that these are the most abundant isoforms in the lung lining fluid [29], we hypothesized that T-cad might also be contributing to the ability of exogenously administered adiponectin to ameliorate allergic airways responses in mice [17], [18]. Consequently, we expected to observe greater effects of OVA challenge in T-cad^−/−^ versus WT mice. Instead, the opposite occurred: allergen-induced responses were reduced in T-cad^−/−^ versus WT mice (Figures 1,3,4,5).

We considered two potential explanations for this result. First, T-cadherin has properties in addition to adiponectin binding. It induces cell de-adhesion and promotes directed cell migration in vascular cells [49]. In endothelial cells, T-cad stimulates proliferation, migration, and survival under conditions of oxidative stress and promotes angiogenesis [49], [50], [51], [52]. T-cad also causes downregulation of surfactant protein D secretion in lung A549 cells [24]. These functions, rather than the ability of T-cad to bind adiponectin, could be responsible for the protective effect of T-cadherin deficiency. To test this hypothesis we allergen sensitized and challenged Adipo^−/−/^T-cad^−/−^ mice and observed that combined deficiency in adiponectin and T-cad reversed the effects of T-cad deficiency alone (Figures 1, 3, 4, 5). Thus, the presence of adiponectin was necessary to elicit the beneficial effects of T-cad deficiency, indicating that the adiponectin binding properties of T-cad were likely involved in the responses observed.

The second explanation is that reductions in allergic airway responses in T-cad^−/−^ mice are caused by elevated serum adiponectin. Serum adiponectin was increased in T-cad^−/−^ mice (Figure 2A, C), consistent with previous observations [26], [27], [28], [29]. T-cad sequesters adiponectin to the endothelium and the heart, and thus serves as an adiponectin repository [26], [28]. Consequently, T-cad deficiency delivers this adiponectin back into the circulation. Thus, the T-cad^−/−^ mice resemble mice given exogenous administration of adiponectin, which are likewise protected from allergic airways disease [17]. Elevated blood adiponectin induced by T-cad deficiency may act to inhibit allergic responses via AdipoR1 and/or AdipoR2, which have been shown to mediate the insulin sensitizing and anti-apoptotic effects of adiponectin [53], [54]. Alternatively, adiponectin may act in a non-receptor mediated fashion. For example, adiponectin interacts directly with growth factors [55] and with other immunomodulatory factors, including thrombospondin-1 [23] and calreticulin [56].

Despite substantial increases in serum adiponectin in T-cad^−/−^ versus WT mice (Figure 2A, C), BAL adiponectin was not different (Figure 2B, D). Further, OVA caused a significant increase in BAL adiponectin in both WT and T-cad^−/−^ mice (Figure 2B, D and Figure 6H), whereas serum adiponectin was not affected (Figure 2A, C and Figure 6G). It is likely that OVA-induced increases in BAL adiponectin are the result of increases in lung alveolar/capillary permeability, since OVA also induced increases in total BAL protein (data not shown). Marked increases in BAL adiponectin are also observed in mice exposed to ozone [57], which also increases lung permeability. The observation that blood but not BAL adiponectin was affected by T-cadherin deficiency suggests that extrapulmonary or intravascular targets of adiponectin might contribute to the observed effects of T-cadherin deficiency. For example, it is conceivable that dendritic cell transit from the lungs to the lymph nodes, or lymphoid tissue development is affected by elevated blood adiponectin in T-cad^−/−^ mice. However, such effects would be expected to impact B cell responses, and OVA-induced increases in IgE were not affected by T-cadherin deficiency (Figures 3D, E). Bone marrow derived dendritic cells do have the capacity to respond to adiponectin [58], and T cells do express adiponectin receptors [59], but our data (Figure 8) indicate that adiponectin does not affect dendritic cell presentation of OVA to T-cells, although the background strain of the mice used for the dendritic cell/T-cell co-cultures was Balb/c not C57BL/6. Konter *et* *al*. recently reported that circulating adiponectin acting on endothelial cells is important for limiting lung inflammatory responses to intratracheally administered endotoxin [27]. Adiponectin has been shown to inhibit TNFα stimulated ICAM, VCAM, and E-selectin expression on cultured endothelial cells [14]. Hence, in T-cad^−/−^ mice, increased circulating adiponectin might also be important for limiting endothelial cell expression of adhesion molecules necessary for migration of immune and inflammatory cells from the blood into the lungs. Consistent with this hypothesis, lymphocytes in the airspaces and BAL concentrations of IL-13 and IL-17, cytokines typically derived from lymphocytes, were reduced in T-cad^−/−^ mice (Figures 1,3,4).

Despite the marked improvement in allergic airways responses observed when blood adiponectin is elevated by exogenous administration [17] or when blood adiponectin was elevated endogenously consequent to T-cad deficiency (Figures 1, 3, 4, 5), we observed little impact of adiponectin deficiency alone on allergic airways responses (Figure 6). Under conditions of reduced adiponectin binding protein expression such as after acute OVA challenge (Figure 7), normal adiponectin concentrations may be insufficient to evoke signal transduction, whereas elevated adiponectin levels in T-cad deficient mice or mice given exogenous adiponectin may be sufficient to induce signaling. Medoff *et* *al* also reported little impact of adiponectin deficiency on allergic airways inflammation when they used an *acute* OVA aerosol challenge protocol similar to that employed here, although allergic airways responses were augmented in Adipo^−/−^ mice following *chronic* intranasal OVA challenge [19]. Those authors suggested that the milder inflammation induced in the chronic model might make it easier for effects of endogenous adiponectin to be observed. Indeed, using the same *chronic* intranasal OVA challenge as Medoff et al employed [19], we observed no change in AdipoR1 or AdipoR2 following OVA challenge (unpublished observations), suggesting that this milder inflammation should allow normal endogenous adiponectin concentrations to evoke effective signal transduction. In any event, our data suggest that effects of adiponectin can be uncovered in the acute OVA model by elevating the blood adiponectin levels via T-cad deficiency: T-cad^−/−^ mice lacking adiponectin had increased responses to OVA compared to T-cad^−/−^ mice that did have adiponectin (Figures 1, 3, 4, 5). Reports from several large genome wide association studies indicate that variation in serum adiponectin concentrations among humans is in part explained by SNPs in the promoter of the T-cad gene [60], [61]. Our data suggest that the T-cad genotype should also be considered in studies evaluating the importance of adiponectin for human asthma.

In conclusion, T-cad does not mediate the protective effects of adiponectin. Instead, mice lacking T-cadherin have reduced allergic airways disease. This reduction is dependent on adiponectin, and likely occurs consequent to elevated serum levels of adiponectin acting on other adiponectin signaling pathways such as AdipoR1 or AdipoR2.
